# Prevalence of long COVID complaints in persons with and without COVID-19

**DOI:** 10.1038/s41598-023-32636-y

**Published:** 2023-04-13

**Authors:** Karin Magnusson, Aleksandra Turkiewicz, Signe Agnes Flottorp, Martin Englund

**Affiliations:** 1 Norwegian Institute of Public Health, Cluster for Health Services Research, Sandakerveien 24C, 0473 Oslo, Norway; 2 Faculty of Medicine, Department of Clinical Sciences Lund, Orthopaedics, Clinical Epidemiology Unit, Lund University, Lund, Sweden; 3 Department of General Practice, Institute of Health and Society, University of Oslo, Oslo, Norway

**Keywords:** Epidemiology, Public health, Signs and symptoms, Fatigue, Infectious diseases, Viral infection, Immunology, Infectious diseases, Viral infection

## Abstract

We studied the prevalence and patterns of typical long COVID complaints in ~ 2.3 million individuals aged 18–70 years with and without confirmed COVID-19 in a Nation-wide population-based prospective cohort study in Norway. Our main outcome measures were the period prevalence of single-occurring or different combinations of complaints based on medical records: (1) Pulmonary (dyspnea and/or cough), (2) Neurological (concentration problems, memory loss), and/or (3) General complaints (fatigue). In persons testing positive (n = 75 979), 64 (95% confidence interval: 54 to 73) and 122 (111 to 113) more persons per 10 000 persons had pulmonary complaints 5–6 months after the test compared to 10 000 persons testing negative (n = 1 167 582) or untested (n = 1 084 578), respectively. The corresponding difference in prevalence of general complaints (fatigue) was 181 (168 to 195) and 224 (211 to 238) per 10 000, and of neurological complaints 5 (2 to 8) and 9 (6–13) per 10 000. Overlap between complaints was rare. Long COVID complaints were only slightly more prevalent in persons with than without confirmed COVID-19. Still, long COVID may pose a substantial burden to healthcare systems in the future given the lasting high incidence of symptomatic COVID-19 in both vaccinated and unvaccinated individuals.

## Introduction

Several studies have reported persistent symptoms following SARS-CoV-2 infection among non-hospitalized individuals, also known as post COVID-19 condition, or long COVID. Such persisting symptoms or complaints are reported to cover a broad range of bodily systems even in persons with mild initial disease, with complaints of general, pulmonary and neurological character being described as the most prevalent^1^. However, their occurrence over time is poorly documented in observational studies, and thus, long COVID or post COVID-19 condition has to date not yet been sufficiently detailed in the existing literature. As SARS-CoV-2 now have affected, or will affect, major parts of both the vaccinated and unvaccinated populations, more people may experience one or several long COVID complaints.

The most commonly reported long COVID complaints to date include shortness of breath and cough (pulmonary complaint), impaired concentration, memory loss or so-called “brain fog” (neurological complaint) and fatigue (general complaint) as well as altered smell or taste^1^. As an example, Lund et al. reported that 8983 non-hospitalized individuals with COVID-19 had a higher prevalence of shortness of breath, cough, and fatigue after COVID-19 than 80 894 non-hospitalized individuals without COVID-19^2^. Fatigue, shortness of breath and brain fog were also reported to be prevalent in population-based studies in Italy and the United States^3,4^. The time frame for having persistent symptoms following COVID-19 is less well studied. In three register-based studies we have recently reported that the duration of an increased number of doctor visits in primary care varies between one and six months, depending on age and sex, with the causes for the visits being restricted to respiratory and general complaints at least in non-hospitalized individuals^5–7^.

To what extent the most reported long COVID complaints are overlapping, and how they develop alone or in combination over time in the vast majority with mild COVID-19 (non-hospitalized), is currently unknown. Studying long-term outcomes in cases with mild disease becomes increasingly important considering that mild, symptomatic disease is common even in vaccinated individuals^8^. Thus, improved knowledge of the course of potential post COVID-19 condition or long COVID may be used to develop a better clinical examination and/or better management for a growing number of patients.

Thus, our aim was to gain new insights into the question of whether and how the post COVID-19 condition or long COVID exists, i.e. how it evolves over time, and how complaints like those experienced in such conditions evolve over time in a general population without COVID-19. In doing so, we focused on general, pulmonary, and neurological complaints, which are the most agreed on persistent complaints reported after COVID-19^1,9^. We used similar approach as reported in a previous study of definitions of early knee osteoarthritis^10^.

## Methods

In this prospective cohort study, we used data from the Norwegian Emergency Preparedness Register^11^. The register includes data from all testing for SARS-CoV-2 (polymerase chain reaction tests—PCR) in Norway from the beginning of the pandemic, all medical records from primary care (used here: general practitioners and emergency wards) and specialist care (used here: for exclusion of hospitalized individuals and for calculation of the number of comoribidities). It also includes data on all vaccine doses for COVID-19 for all individuals as well as background characteristics such as age, sex and country of birth.

Our inclusion criteria were persons living in Norway on August 1st 2020, age between 18 and 70 years, who were tested (with a positive or negative result), or not tested for SARS-CoV-2 in between August 1st 2020 and August 1st 2021 without being hospitalized from − 2 to + 14 days from the test date (or a hypothetical test date for the untested), and without having a diagnostic code of any of the included operational definitions of post-covid symptoms or complaints (general practitioner or emergency ward), from six months prior to the test date for SARS-CoV-2 to the beginning of the week of testing. In this way, we allowed for prevalent complaints/healthcare use in relation to testing but not in relation to pre-test complaints/healthcare use. We also avoided the selection bias that may arise because of routine testing prior to specialist healthcare use^5^.

Test criteria during our study period included everyone having symptoms or being a close contact to a person with confirmed or suspected SARS-CoV-2 infection. We could not include antigen testing, however every positive antigen test was required to be followed up by a PCR test (of which we captured all in the current study).

Based on previous findings of increased health care use for 1 to 6 months after positive tests^5–7^, we included follow-up data from primary care for 4 different time points during follow-up: baseline (test week), 2, 4 and 6 months after the test date. All contacts that occurred during the time passing between the time points were included in the latter time point (i.e. all contacts from week 1 to week 7 were included in week 8 together with the contacts in week 8, to allow for clustering of contacts for different causes over time). We required at least a six months follow-up time, i.e. the few persons dying or emigrating during follow-up were excluded ensuring everyone could be observed throughout the entire study period.

The Ethics Committee of South-East Norway confirmed (June 4th 2020, #153,204) that external ethical board review was not required. The data sources (The emergency preparedness register for COVID-19 (Beredt C19)) were established and handled in accordance with the Health Preparedness Act §2–4^11^. All methods were carried out in accordance with relevant guidelines and regulations. No informed consent from participants was required since our study was based on routinely collected register data.

### Study groups

The study sample was dividied into three mutually exclusive study groups according to their test status, as previously described^7^:

- Persons testing positive for SARS-CoV-2, including everyone with one or more positive tests in the inclusion period. In the rare cases of several tests with a positive result, we chose the first one. Persons whose first positive test fell outside the inclusion period were excluded.
- Persons testing negative for SARS-CoV-2, including everyone with one or more negative tests in the inclusion period. If there were several tests with a negative result in- or outside the inclusion period, we randomly chose one of the tests. Persons whose randomly drawn negative test fell outside the inclusion period were excluded. In this way, frequent and less frequent testers had the same probability to have a test during the inclusion period.
- Untested persons, including everyone who were never tested for SARS-CoV-2 neither in- or outside the study period, and who were assigned a random, hypothetical test date falling in the inclusion period (equal probability for each date).

### Outcomes: operational definitions of long-covid

We studied medical symptoms and complaints reported from primary care that were diagnosed by general practitioners and medical doctors at emergency wards that fell in the following categories based on International Classification of Primary Care (ICPC-2) codes:

- Pulmonary complaints: shortness of breath/dyspnea (R02), cough (R05)
- Neurological complaints: impaired concentration, memory problems or brain fog (P20)
- General complaints: fatigue (A04, A05, A29)

Based on these categorizations, we made seven operational definitions of typical long COVID complaints, i.e. we studied the single categories and all combinations of the three categories as separate outcomes, for each study time point: (1) Pulmonary complaints, (2) Neurological complaints, (3) General complaints, (4) Pulmonary + neurological complaints, (5) Pulmonary + general complaints, (6) Neurological + general complaints, and (7) Pulmonary + neurological + general complaints.

In this way, outcomes (1) to (3) were studied as outcomes occurring on their own („single complaints “), whereas outcomes (4) to (7) were studied as outcomes occurring in combination („combined complaints “). The operational definitions were made arbitrarily in the lack of established definition of a post COVID-19 or long COVID symptom or complaint, however they were in large extent in accordance with the recently agreed on WHO-definition for the post COVID-19 condition^9^.

Medical recording to the National registries is mandated by law in Norway, ensuring no missing outcome data in our study. Norwegian health register data have been demonstrated to have high validity and reliability in a small comparative study of medical journal notes and medical records^12^, i.e. they may be used for studying patterns of health care use and complaints leading to health care use. Because seeking healthcare was a requirement for our definitions, we allowed for up to two months to pass for the definitions to overlap as described above.

### Statistical analyses

First, at baseline, 2, 4 and 6 months we studied the prevalence of the typical long COVID complaints, according to the seven operational definitions above, in the group testing positive for SARS-CoV-2, in the group testing negative and in the untested group. To take into account the dependence of the data at each time point for each person, we determined the groupwise point prevalence and its 95% confidence intervals (CI) of each outcome at each time points from a logistic regression model with robust standard errors (clustered on patient). Second, to compare the prevalence of the complaints at baseline and the follow-ups (2, 4 and 6 months) between the exposure groups, we used a logistic regression model. In the model, the group (testing positive vs negative and testing positive vs untested), the time points (0, 2, 4 and 6 months), and their interaction were included as fixed effects, while the patient was included as a random effect. All regression analyses were adjusted for age, sex, education level (in four categories: no education, primary school, upper secondary school or college/university), country of birth (Norway vs abroad), the number of comorbidities (0, 1 or 2 or more, as based on risk conditions for severe COVID-19 defined by an expert panel in ethics and prioritisation, with data identified in data from the Norwegian Patient Register)^13^, the number of vaccine doses (0, 1 or 2 or more) and calendar month with year as potential confounders. A separate model was fitted for each outcome.

Further, to assess whether the estimated group differences could be affected by previous history of any of the definitions, we repeated the analyses with adjustment for medical records from general practitioner and/or the emergency ward that were indicative of pulmonary, neurological and/or general complaints during 2017–19, using diagnostic codes as described above (0 (absent) vs 1 (present) for the complaint in question).

To further illustrate the changes in prevalence and overlap of the outcome definitions over time in the two exposure groups, we used proportional Venn-diagrams. All analyses were performed in Stata MP v. 17.

## Results

**Figure 1:**
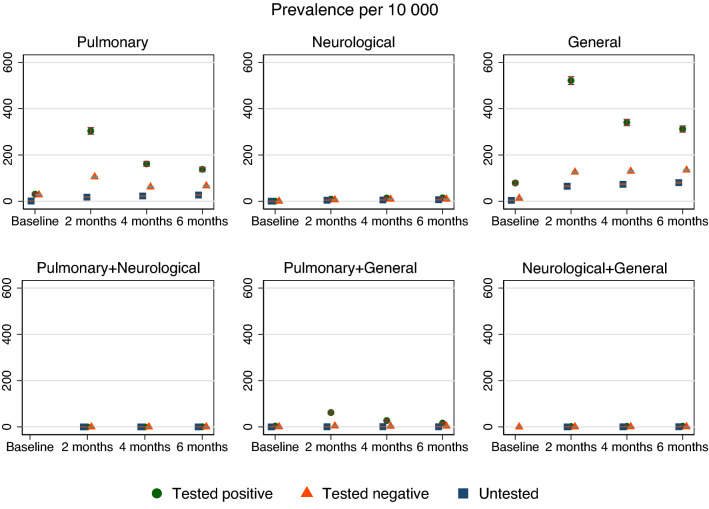
The adjusted prevalence in percent and 95% confidence interval for different long COVID complaints at baseline, 2, 4 and 6 months followup for persons testing negative and positive for SARS-CoV-2. Missing circles, triangles or squares indicate no observation for the group in question. The prevalence of pulmonary + neurological + general complaints could not be plotted due to very few observations.

Of in total 3 720 465 persons living in Norway on August 1st 2020, 2 348 831 fulfilled our inclusion criteria, of which n = 76 194 tested positive for SARS-CoV-2 and n = 1 173 221 tested negative, with 1 099 416 being untested (Supplementary (S)-Fig. 1). Among persons testing positive, 40 (0.05%) died and 175 (0.2%) emigrated within the six months follow-up, leaving 75 979 persons testing positive for our analyses. Among persons testing negative, 676 (0.06%) died and 4693 (0.4%) emigrated within the six months follow-up, leaving 1 167 582 persons testing negative for our analyses. Among untested persons, 693 (0.06%) died and 14 145 (0.1%) emigrated within the six months follow-up, leaving 1 084 578 untested persons for the anlayses.

Persons testing positive were younger, had fewer comorbidities and were less often vaccinated than persons testing negative and the untested group (Table 1). They also had a higher prevalence of seeking medical care for general complaints during 2017–19 than the comparison groups, yet no signs of seeking more care for all the three included complaints (Table 1).

**Table 1:** Descriptive characteristics.

	Testing positive for SARS-CoV-2	Testing negative for SARS-CoV-2	Untested for SARS-CoV-2
	N = 75 979	N = 1 167 582	N = 1 084 578
Age, mean (SD)	36.7 (13.6)	40.2 (14.1)	48 (14.7)
Women, n (%)	34 772 (45.7)	577 098 (49.4)	463 522 (42.7)
Primary school, n (%)	22 549 (29.7)	230 358 (19.7)	252 154 (23.3)
Upper secondary school, n (%)	24 520 (32.3)	421 400 (36.1)	443 971 (40.9)
College/university, n (%)	21 920 (28.9)	456 411 (39.1)	298 404 (27.5)
Born in Norway, n (%)	45 958 (60.5)	940 179 (80.5)	854 142 (78.8)
One comorbidity, n (%)	7307 (9.6)	128 701 (11.0)	162 721 (15.0)
≥ 2 comorbidities, n (%)	242 (0.3)	4288 (0.4)	7526 (0.7)
One vaccine dose, n (%)	4524 (5.6)	110 330 (9.4)	142 276 (13.1)
≥ 2 vaccine doses, n (%)	542 (0.7)	31 192 (2.7)	58 767 (5.4)
Previous pulmonary complaints*, n (%)	7695 (10.1)	123 914 (10.6)	92 985 (8.6)
Previous neurological complaints*, n (%)	547 (0.7)	8045 (0.7)	6899 (0.6)
Previous general complaints*, n (%)	12 621 (16.6)	172 555 (14.8)	106 533 (9.8)
Nr of previous visits for pulmonary, neurological and/or general complaints^+^, single occurring or in combination, median (IQR)	0 (0–1)	0 (0–1)	0 (0–1)

All characteristics measured prior to test.

*Visited general practitioner or emergency ward at least once in 2017–19. ^+^General practitioner and/or emergency ward, 2017–19.

### Prevalence of single complaints

The prevalence of the single complaints increased from baseline to 2 months for all three groups of complaints, yet decreased from 2 to 6 months for pulmonary and general complaints. There was a lower prevalence and a smaller increase over time for neurological complaints (Fig. 1).

Persons testing positive had more prevalent pulmonary and general complaints than persons testing negative and untested persons, with the difference in prevalence being the largest at 2 months after the test week, before decreasing at 4 months and 6 months followup (Fig. 1, Table 2). Group differences in neurological complaints were smaller than group differences in pulmonary and general complaints, and generally stable or slightly increasing over time, from 2 months to 4 and 6 months followup (Fig. 1, Table 2).

**Table 2:** The differences between the group testing positive and the comparison groups in prevalence of different long COVID complaints over time.

	Test positive versus test negative				Test positive versus untested			
	Baseline	2 months	4 months	6 months	Baseline	2 months	4 months	6 months
One complaint								
Pulmonary	2	177	89	64	32	316	154	122
	− 3 to 7	164 to 190	79 to 98	55 to 73	26 to 39	299 to 333	141 to 166	111 to 133
Neurological	NE	3	5	5	NE	5	9	9
		1 to 5	2 to 8	2 to 8		3 to 8	6 to 13	6 to 13
General	68	406	216	181	73	442	259	224
	60 to 76	388 to 423	202 to 230	168 to 195	65 to 80	425 to 459	245 to 272	211 to 238
Combinations of two complaints								
Pulmonary + general	3	54	23	12	4	70	30	18
	0 to 5	47 to 60	18 to 27	8 to 16	1 to 7	60 to 79	24 to 36	13 to 22
Neurological + general	NE	1	2	3	NE	1	2	3
		0 to 2	0 to 4	1 to 5		0 to 2	1 to 4	1 to 4

Estimates are group differences in prevalence per 10 000 persons in the respective groups, with 95% confidence intervals, representing the group testing positive minus the group testing negative and the untested group, in separate analyses. Group differences for pulmonary + neurological and for all three combinations (pulmonary + neurological + general) could not be estimated (NE) due to too few observations for test positive.

The differences imply that an additional (approximal) 50 to 250 persons per 10 000 persons with COVID-19 would visit their primary care doctor and get an ICPC-2 code for pulmonary or general complaints at 6 months after positive test, compared to 10 000 persons without COVID-19 (testing negative or untested). Similarly, the increase for neurological complaints corresponded to 5 to 10 additional persons visiting medical care per 10 000 persons with COVID-19. The estimates and interpretations were similar when we repeated the analyses with adjustment for prevalent complaints in 2017–19, although the bewteen-group differences were slightly decreased when using the persons testing negative as comparison group (S-Table 2).

### Combinations and overlap of long COVID complaints over time

Combinations of two and three long COVID complaints were less prevalent than single long COVID complaints for all groups (S-Table 1, Figure 1). However, persons testing positive had a higher crude prevalence of combined complaints than persons testing negative and untested persons (S-Table 1). Due to the low numbers, between-group differences could only be estimated for a few combinations. Pulmonary + general complaints showed a decreasing prevalence from 2 to 6 months, whereas the estimated prevalence of neurological + general complaints was stable and/or slightly increasing from 2 to 6 months (Fig. 1, Table 2). The group differences imply that an additional 10 to 20 persons per 10 000 persons with COVID-19 would visit their primary care doctor and get an ICPC-2 code for combined pulmonary and general complaints at 6 months after positive test, compared to 10 000 persons without COVID-19 (testing negative or untested).

There were few observations for pulmonary + neurological complaints and we did not attempt to estimate group differences (S-Table 1). Further, we observed fewer than five persons having all complaints combined (pulmonary + neurological + general) for all time points, i.e. we neither plotted, nor estimated the group differences (S-Table 1, Figure 1, Table 2).

**Figure 2:**
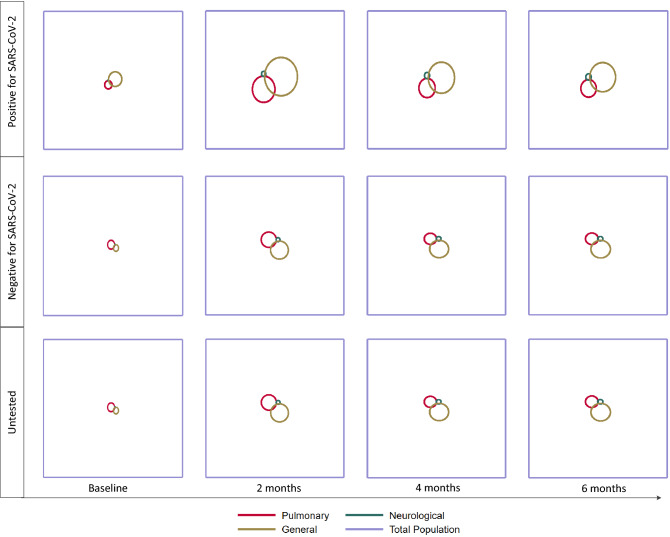
Proportions and intersections of complaints in the population in the group testing positive, the group testing negative and the untested group at baseline, 2, 4 and 6 months follow-up.

The small overlap and higher prevalence of complaints in persons testing positive than negative and in untested were visualized in proportional Venn-diagrams (Fig. 2). There were no signs of an increasing overlap over time, and the largest overlap was observed at 2 months for persons testing positive having pulmonary and general complaints (Fig. 2). This overlap was visually lower at 6 months (Fig. 2).

## Discussion

In the present prospective cohort study of 2.3 million persons with and without COVID-19, we found that the frequency of typical long COVID complaints was around 5 to 250 per 10 000 persons higher in subjects having tested positive for COVID-19 vs those testing negative and vs the general population without COVID-19. Long COVID fatigue, pulmonary and neurocognitive complaints were usually single-occurring and not seen in combination. Thus, complaints that are often regarded as typical long COVID complaints may also be naturally prevalent complaints, irrespective of initial disease. The high incidence of COVID-19 is still a source of concern as the absolute number of patients with these post-infection complaints is high.

### Comparison to previous studies

Although several studies have assessed the risk of different outcomes after COVID-19 usually based on matching of individuals with and without diagnostic codes of the disease^1,4,14^, we could find no study exploring the prevalence, clustering and timing of complaints that are typically reported as long COVID complaints in a general population. In that regard, our study sheds new light compared to recent studies comparing complaints after COVID-19 to complaints after influenza in healthcare seeking individuals ^4^. Whereas we found risk increases of 64 (54 to 73), 181 (168 to 195) and 5 (2 to 8) per 10 000 for persons testing positive vs negative for SARS-CoV-2, for pulmonary, general and neurological complaints at 6 months, respectively, the corresponding risk increases for persons with a diagnostic code of COVID-19 vs diagnostic code of influenza were 440 (390 to 488), 265 (222 to 308) and 118 (89 to 148) per 10 000, respectively. The somewhat lower estimates in our study vs previous studies may be explained by differences in inclusion criteria. Whereas previous studies included unvaccinated persons with COVID-19 vs. influenza as registered in diagnostic codes in primary or specialist care (and thus were regarded to be more severely affected by the two diseases)^4^, we could here study everyone who were tested (positive or negative) or untested for SARS-CoV-2 in the general population, and thus including both symptomatic, asymptomatic, vaccinated and unvaccinated, healthcare- and non-healthcare-seeking individuals in our exposure variables. The included persons in our study were non-hospitalized and thus were regarded to be relatively mildly affected.

Together, these studies suggest that pulmonary complaints and fatigue are slightly more prevalent at 6 months after testing positive than after testing negative or not being tested in mildly affected, and also slightly more prevalent 6 months after influenza for the more severely affected^4^. The post COVID-19 condition as recently defined by WHO ^9^ thus likely affects relatively few and might come in different phenotypes. For example, neurological complaints and combined complaints were less prevalent both in our and previous studies^4^ and there were fewer group differences than for pulmonary and general complaints, at all time points. To our knowledge, our study represents one of the first attempts to estimate the prevalence of long COVID complaints in a general population using definitions largely in accordance with the newly agreed on WHO-definition^9^. Still, our data were based on medical records from healthcare services and will need to be confirmed in studies based on patient-reported or clinical data. Such data are more sensitive, probably finding more overlap and combination of long COVID complaints than reported in the current study^15,16^. Finally, it should be noted that although we found low prevalence and small between-group differences in our study, the absolute burden of long COVID complaints may be high. Provided that almost everyone, even vaccinated individuals, can expect to catch the virus and experience some level of symptoms^8^, our findings of 5 to 250 additional persons per 10 000 experiencing long COVID symptoms or complaints may be a source of concern when millions of people are infected simultaneously.

### Interpretation, relevance and future research directions

The current study adds to the understanding of how the most typical long COVID complaints evolve and cluster over time as well as how large a burden it will give on the healthcare services when many are infected with SARS-CoV-2. Besides shedding light on healthcare visits and the development of symptoms related to post COVID-19 condition in a research perspective, our study may be of relevance to clinicians and their patients, policymakers and researchers. As such, the knowledge drawn from the current study should be seen in light of our study period (August 1st 2020 to August 1st 2021), which was dominated by the Wuhan and Alpha SARS-CoV-2 variants and which was characterized by the beginning of mass vaccination. In persons testing positive, 6% had received one or more vaccine doses (Table 1). Thus, our findings may need to be replicated for later pandemic periods, for example for the current pandemic omicron wave, which is characterized by a high vaccination coverage. For the current pandemic period, our recent study^17^ suggest that persons with omicron and delta both had similarly increased risks of post COVID-19 fatigue and shortness of breath for up to 126 days after being tested when adjusted for vaccination status. If these observations are correct, i.e. if both delta and omicron are comparable to each other and earlier SARS-CoV-2 variants in terms of long COVID complaints, our reported risk differences are applicable to the current situation (as long as omicron is dominant).

Other relevant future research directions following our study include the effectiveness of booster dose vaccination on long COVID complaints^18^, the characteristics of persons who develop post COVID-19 condition and subsequent excess healthcare use, as well as what (combinations of) characteristics are predictive for such outcomes (e.g. men vs women, high vs low education, virus variant, vaccination etc.). The development of prediction models may aid in the identification of what are the modifiable predictive factors for long COVID complaints, which can subsequently be used by clinicians and public health workers for preventive aims. Finally, more knowledge of post-viral complaints after mild COVID-19 vs after comparable respiratory tract infections in a general population is warranted.

### Strengths and limitations

An important strength of our study was the inclusion of all persons tested and not tested for SARS-CoV-2 in Norway, vaccinated and unvaccinated, in a period with unchanged test criteria and the ability to link these data with recent and complete medical records (up to February 2022). Further, we could include two comparison groups. Persons testing negative (or positive) were likely tested for a reason, for example covid-like symptoms or being a close contact to someone with confirmed COVID-19, but there may also have been a substantial proportion of persons free of symptoms. In that regard, the group of persons being tested (with positive or negative result) may consist of particularly health-conscious persons. Health consciousness and propensity to seek first-line care might explain the more frequent use of primary care prior to infection in the infected group (Table 1). The untested comparison group, in contrast, is likely more representative of the general population without conditioning on current symptoms or test activity. It is reassuring that estimates of between-group differences were of similar magnitudes or slightly higher when using the group of untested individuals as reference. Another strength is that our findings are representative for countries providing equal access to healthcare services, including equal access to PCR testing for SARS-CoV-2, to all inhabitants (at least countries with similar rates of COVID-19 infections). The lack of data on antigen testing is not likely to have affected our findings as its use was limited in Norway during our study period (and positive antigen test performed during our study period should always be followed up with PCR testing). However, the generalizability of our results to later pandemic waves need caution.

Several limitations should be mentioned. First, the use of medical records in primary care as a basis of definitions of long COVID complaints may underestimate their true prevalence. Although medical records from primary care have been found to have a high validity and reliability in Norway^12^, patients may seek medical care only if their complaints are severe enough. Further, medical doctors may not use all diagnostic codes that are relevant when a patient presents with multiple complaints, i.e. the true overlap may be higher than reported. We avoided the inclusion of specialist medical care records here, as these had the same prevalence in persons testing positive vs negative for SARS-CoV-2 in an earlier study of the same study period ^5^. Finally, our definitions of long COVID symptoms and complaints were based on medical assessments and categorization of general practitioners and medical doctors at emergency wards. In that regard, we studied health-seeking behavior rather than health, and our operational definitions of long COVID complaints can only be regarded as proxies for health. Thus, we cannot exclude the possibility that having tested positive may lead to a higher propensity to seek care for symptoms post their infection as compared to persons having tested negative.

In conclusion, we find that long COVID complaints were only slightly more prevalent in persons with than without COVID-19 in a general population of 2.3 million persons. Our observations suggest that the post COVID-19 condition affects few however may still pose a substantial burden to healthcare systems given the widespread transmission of SARS-CoV-2.

## Supplementary Information

Supplementary Information.

## Data Availability

The study method and statistical analyses are all described in detail in the Methods chapter and throughout the paper. Individual-level data of patients included in this paper after de-identification are considered sensitive and will not be shared. However, the individual-level data in the registries compiled in Beredt C19 are accessible to authorized researchers after ethical approval and application to "helsedata.no/en" administered by the Norwegian. Directorate of eHealth. Data requests may be sent to "service@helsedata.no.
