# Supplementary material for: Prevalence of long COVID complaints in persons with and without COVID-19
Source: Sci Rep. 2023 Apr 13;13:6074. doi: 10.1038/s41598-023-32636-y (PMC10100609; doi:10.1038/s41598-023-32636-y)
Supplement: Supplementary file 1 — Supplementary Information. [file 41598_2023_32636_MOESM1_ESM.pdf]

Supplementary file for the paper

Prevalence of long COVID complaints in persons with and without COVID-19 in primary care

By Karin Magnusson, Aleksandra Turkiewicz, Signe Agnes Flottorp and Martin Englund, 2023.

Table of contents (brief titles):

S-Figure 1. Flow chart.	**p. 2**
S-Table 1. The overlap between long-covid definitions.	**p. 3**
S-Table 2. Group differences (positive vs negative and positive vs untested), adjusted for previous prevalent complaints	**p. 4**

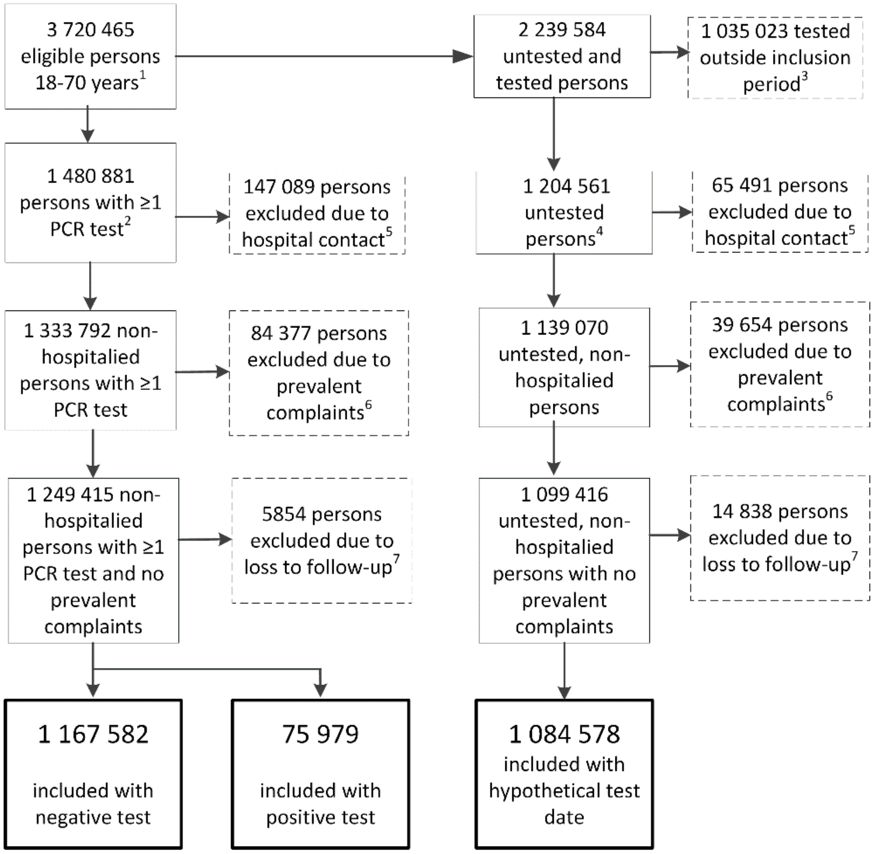

[1] Alive and living in Norway on August 1 2020
[2] During inclusion period (August 1 2020 to August 1 2021)
[3] Between February 1 and July 31 2020, and/or between August 2 and January 31 2022
[4] Assigned a random, hypothetical test date in study period
[5] All-cause, inpatient or outpatient, from -2 to +14 days from (hypothetical) test date
[6] One or more records in primary care with any of the operational long-covid definitions, from six months prior to (hypothetical) test date, to (hypothetical) test date
[7] Death or emigration during the six months follow-up

Supplementary Figure 1. Flow chart showing eligible, excluded and included persons.

Supplementary Table 1. The overlap between the prevalence of seven long COVID definitions at baseline, 2, 4 and 6 months.

	Pulmonary	Neurological	General	Pulmonary + neurological	Pulmonary + general	Neurological + general	All
**Baseline**							
Testing positive	186 (0.2)	<5 (0.007)	602 (0.8)	<5 (0.007)	27 (0.04)	<5 (0.007)	<5 (0.007)
Testing negative	2937 (0.3)	39 (0.003)	1610 (0.1)	<5 (0.0004)	81 (0.007)	8 (0.0007)	<5 (0.0004)
Untested	148 (0.01)	32 (0.003)	414 (0.04)	<5 (0.0005)	8 (0.0007)	<5 (0.0005)	<5 (0.0005)
**2 months**							
Testing positive	1850 (2.4)	69 (0.09)	3939 (5.2)	<5 (0.007)	419 (0.6)	12 (0.02)	<5 (0.007)
Testing negative	11061 (0.9)	748 (0.06)	15329 (1.3)	13 (0.001)	465 (0.04)	54 (0.005)	<5 (0.0004)
Untested	2203 (0.2)	510 (0.05)	6754 (0.6)	<5 (0.0005)	66 (0.006)	37 (0.003)	<5 (0.0005)
**4 months**							
Testing positive	976 (1.3)	102 (0.13)	2565 (3.4)	<5 (0.007)	183 (0.2)	19 (0.03)	<5 (0.007)
Testing negative	6467 (0.6)	1029 (0.09)	15770 (1.4)	11 (0.0009)	323 (0.03)	93 (0.008)	<5 (0.0004)
Untested	2764 (0.3)	592 (0.05)	7611 (0.7)	<5 (0.0005)	102 (0.009)	45 (0.004)	<5 (0.0005)
**6 months**							
Testing positive	812 (1.1)	105 (0.1)	2265 (3.0)	<5 (0.007)	109 (0.1)	23 (0.03)	<5 (0.007)
Testing negative	6744 (0.6)	1063 (0.09)	15799 (1.4)	26 (0.002)	374 (0.03)	84 (0.007)	<5 (0.0004)
Untested	3159 (0.3)	683 (0.06)	7960 (0.7)	6 (0.0006)	88 (0.008)	57 (0.005)	<5 (0.0005)

Data are numbers with percentages for the group testing positive (n=75 979), the group testing negative (n=1 167 582) and the untested group (n=1 084 578).

Supplementary Table 2. The differences between the group testing positive and the comparison groups in prevalence of different long COVID complaints over time, adjusted for prevalent pulmonary, neurological and/or general complaints during 2017-19.

	**Test positive vs test negative**				**Test positive vs untested**			
	**Baseline**	**2 months**	**4 months**	**6 months**	**Baseline**	**2 months**	**4 months**	**6 months**
**One complaint**								
Pulmonary	3<br>-2 to 8	180<br>167 to 193	90<br>80 to 99	65<br>56 to 74	30<br>24 to 35	290<br>274 to 306	140<br>128 to 152	110<br>100 to 121
Neurological	NE<br>NE	3<br>1 to 5	5<br>2 to 8	6<br>2 to 9	NE<br>NE	5<br>2 to 7	8<br>5 to 11	8<br>5 to 11
General	65<br>57 to 73	387<br>370 to 404	203<br>189 to 216	169<br>155 to 182	63<br>56 to 70	386<br>370 to 402	221<br>208 to 233	189<br>176 to 201
**Combinations of two complaints**								
Pulmonary + general	3<br>0 to 6	52<br>46 to 59	22<br>17 to 26	12<br>8 to 15	4<br>1 to 7	64<br>54 to 73	27<br>22 to 33	16<br>12 to 20
Neurological + general	NE<br>NE	1<br>0 to 2	2<br>0 to 3	3<br>1 to 4	NE<br>NE	1<br>0 to 2	2<br>1 to 3	2<br>1 to 4

Estimates are group differences in prevalence per 10 000 persons in the respective groups, with 95% confidence intervals, representing the group testing positive minus the group testing negative and the untested group, in separate analyses. Group differences for pulmunoary+neurological and for all three combinations (pulmonary+neurological+general) could not be estimated due to too few observations for test positive.
